# The Role in Road Traffic Accident and Anxiety as Moderators Attention Biases in Modified Emotional Stroop Test

**DOI:** 10.3389/fpsyg.2019.01575

**Published:** 2019-07-09

**Authors:** Dawid Konrad Ścigała, Elżbieta Zdankiewicz-Ścigała

**Affiliations:** ^1^Institute of Psychology, Faculty of Applied Social Sciences, The Maria Grzegorzewska University, Warsaw, Poland; ^2^Faculty of Psychology, SWPS University of Social Sciences and Humanities, Warsaw, Poland

**Keywords:** emotional Stroop, anxiety, attention biases, road traffic accident, acute stress disorder

## Abstract

**Introduction:**

According to the World Health Organisation, road accidents will be the most common cause of premature death by 2020. According to research, one in every five victims of accidents suffers from acute stress disorder and one in every four suffers from psychological problems up to 1 year after the event, including post-traumatic stress disorder. It was assumed that one of the mechanisms responsible for maintaining excessive arousal or anxiety is a dysfunction in cognitive processes occurring under the guise of selective attention disorders or a deficit in executive control.

**Materials and Methods:**

The research encompassed 157 individuals (a group of victims and perpetrators *N* = 90; *M* = 34.1, *SD* = 10.77; control group *N* = 67; *M* = 34.20, *SD* = 11.16). The participants, tested after road traffic accidents, were patients of Traumatology and Orthopedic wards in Warsaw who had been involved in a road traffic accident up to a month prior to the research. The state of their physical injuries and administered drugs were monitored so that this did not interfere with the tests the participants undertook on computer. In each situation, the decision was made by the doctor responsible for the patient in the hospital ward. The control group comprised people who drive regularly and in 5 years had not been involved in any road traffic incidents. The participants from both groups completed the State–Trait Anxiety Inventory questionnaire on anxiety as a state and as a trait, as well as a modified computerized emotional Stroop test. This new version of the test enables a study of the process of the depth of coding of the stimuli associated with trauma.

**Results:**

The hypotheses were tested with the use of a series of correlation analyses, regression analyses with a stepwise method of entering predictors into the model, and mediation analyses with the use of the A. F. Hayes PROCESS macro. Differences were observed in the declarative level of anxiety as a state and the size of the interference effect depending on the person’s status in the accident. It was discovered that in the group of perpetrators, the longer the interference effect, the lower the declared level of anxiety as a state and they were significantly worse at remembering the stimuli associated with trauma.

**Conclusion:**

Anxiety symptoms in victims and perpetrators of road traffic accidents measured by self-report questionnaires are consistent only among victims. In the case of perpetrators, an accurate measure of disorders is a study with the use of methods enabling the tracking of the functioning of unconscious processes.

## Introduction

Nearly 90% of people experience various kinds of trauma in their lifetime. Approximately 10–15% of people who had a traumatic experience suffer from post-traumatic stress disorder (PTSD) (Benjet et al., 2016). According to the World Health Organisation, road accidents will be the most common cause of premature death by 2020. In European countries, road traffic accidents are the sixth most common cause of disability and the 13 most common cause of death. Moreover, research shows that nearly one in every five victims of accidents may be diagnosed with acute stress disorder (ASD), while one in every four presents psychological problems within a year after the event (Mayou et al., 2001; Bryant et al., 2003; Ścigała, 2013; Dai et al., 2018). It follows from British and American data that road traffic accidents are the most frequent cause of numerous emotional disorders, including PTSD.

Factors conditioning the development of post-traumatic disorders concentrate around the strength and type of the individual’s experiences occurring during the event (van der Kolk, 2000). The experiences depend on the nature and type of traumatic event, its psychological importance, and physical parameters of stimulus situations. Although specific events can as a rule be ordered on such measures as the degree of threat to physical integrity and the intensity of stimulation, the importance of a given event is determined by its subjective perception and the cognitive representation of the traumatic stimuli that develops in the individual’s mind (Shalev, 2009). According to Joseph et al. (1995), it is the elements of the stimuli representation which gets coded in an individual’s memory that are the source of intrusive thoughts and images. Depending on the content and nature of the representation, their processing may be easier or more difficult, and the emotional and physiological reaction of the body is their further consequence. The more fragmentary the representation, the more difficult the process of memory trace consolidation (Dębiec, 2006; Squire et al., 2015). What is essential in stoking fear after a traumatic experience is the lack of a full narration of the experience and storing it in autobiographical memory in a fragmentary form (van der Kolk and Fisler, 1995). That is also why it is important to identify the mechanisms responsible for maintaining excessive arousal or anxiety. According to the assumption made by the authors, one of those mechanisms might be dysfunctions in cognitive processes (Ehlers and Clark, 2000) – from a tendentious perception of the stimuli associated with trauma to a deficit in executive control, measured, among others, by the interference effect in the Stroop test.

Stroop discovered in 1935 that an automated action (e.g., reading a word) disturbs or interferes with the performance of a poorly automated action (naming the color in which the word is written). The original Stroop test was subject to many extensions and modifications. However, it was proved that words which are semantically linked to color (e.g., red and blood) will cause a stronger interference than ones which are not. The conclusion was reached that words might be linked in the mind in semantic networks and an activation of such elements of the network may extend reaction time. Watts et al. (1986) asked people afraid of spiders to name the color of neutral words and words related to spiders (e.g., “spider,” “web,” “cobweb”). Those persons needed much more time to name the color of the words related to spiders than neutral words. In the control group (people not feeling such anxiety) there was no difference in the time of naming both types of words. This way the emotional Stroop test was discovered and began to be used in many studies, among others in research on groups of people with anxiety and post-traumatic disorders.

The fact that the content of emotionally tinged words attracts attention quickly and automatically, which hinders the inhibition of the automated reaction of reading the words, is believed to be responsible for the effect of longer reaction times to emotionally charged words. In consequence, it takes longer to respond to the question about font color. The emotional Stroop test (or task) registers an important type of interference whose control depends on the properties of the emotion – of course, most commonly it is anxiety. The core area of the application of the test are clinical studies to assess – based on the interference degree indicators for words of various content – the degree of emotional disorders resulting from the trauma experienced by a person or the source of the trauma. A word related to the source of the trauma attracts attention to a greater extent and therefore it takes longer to name the font color (Williams et al., 1996). Results obtained in the Stroop test are treated as a measure of “cognitive control over the interfering influence of the automated reaction of reading, hence this method is also used to measure the control of inhibition in a situation of conflict” (Jodzio, 2008, p. 263). Therefore, it is an indicator of the functioning of working memory, concentration ability, or executive control identified with the resistance to interference (Okruszek and Rutkowska, 2013). This explains why the Stroop test found a particular application in research on brain activity during neuroimaging in people with various affective disorders (Whalen et al., 1998; Liu et al., 2015), suffering from schizophrenia (Borkowska, 2006) or anxiety (Williams et al., 1996; Mogg and Bradley, 2018), where the specific characteristics of cognitive deficits indicates a dysfunction in the area of the prefrontal cortex and cingulate cortex (Zysset et al., 2002; Alvarez and Emory, 2004). Results of these studies can be summed up as follows: the more efficiently these brain areas work, the shorter the response times to stimuli associated with trauma in the Stroop test. The poor the functioning of the aforementioned brain areas, the longer the reaction time to an emotogenic stimulus and it can thus be concluded that there are stronger dysfunctions in these brain areas.

Williams et al. (1996) made an exhaustive overview of research concerning the use of the emotional Stroop task in psychopathology and concluded that, to paraphrase, the emotional Stroop task meets the expectations as an instrument to measure the degree to which attention distortion is involved in the maintenance of psychopathology and that does not result from artifact variables. It is sensitive to differences depending on the type of psychopathology and is an indicator of the extent to which the person recovered from the disease after (Williams et al., 1996, p. 22). Beck et al. (2001) undertook research in which the functioning of selective attention was compared in three groups: (a) persons after a road traffic accident who were diagnosed with PTSD and pain; (b) persons after a road traffic accident with general pain but without diagnosed PTSD; and (c) persons without diagnosed anxiety. The researchers diagnosed the level of pain felt by people who had been involved in road traffic accidents. Additionally, the duration of the stay in hospital after the accident was taken into account. The research question concerned the extent to which the interference effect has a specific nature and occurs in response to the stimuli associated with the experienced trauma only, and the extent to which it is a generalized reaction to the stimuli from the category of those affectively negative with regard to road traffic accidents. The authors emphasize that their results confirm simultaneously the universality and individuality of attention disorders in persons with anxiety. Universality regards the fact that selective attention disorders are observed in the processing of negative stimuli in the case of anxiety. Individuality is linked to the fact that the interference effect concerns stimuli associated with a personal trauma. It is interesting that persons with a dual diagnosis showed decidedly longer reaction times in naming both the words associated with an accident and those associated with pain. Generally, in this group there were longer reaction times to all categories of stimuli. In the group without PTSD but with pain this effect was not observed.

In turn Coates (2008) verified the hypothesis concerning the influence of implicit memory on the interference effect in the Stroop test. For this purpose he selected as his research groups the following: (a) persons who had been involved in a road traffic accident, suffered brain damage, and did not remember the accident (15 persons); (b) persons who had been involved in a road traffic accident and retained a memory of the event (13 persons); and, as a control group, (c) patients of an orthopedic ward (15 persons). The participants completed questionnaires to measure ASD and anxiety as a trait and as a state State–Trait Anxiety Inventory (STAI). The researcher demonstrated that there was no difference in the interference effect between the group of persons who had been involved in a road traffic accident and retained a memory of the event and those whose memory of the event was implicit. The author suggests that the emotional Stroop test might be a sensitive instrument for the measurement of the emotional memory of an event and the lack of explicit memory of trauma does not have to have a protective effect on the individual in such a way that post-traumatic disorders will not occur in the person. The fact that both groups of victims of accidents presented the same pattern of reaction to the words in the Stroop test indicates that the effects concern the injury sustained and not the stay in hospital.

Equally interesting are the results of an extremely ingenious study conducted by Thomas and Fremouw (2009). The question the researchers asked themselves was as follows: how will persons who know what post-traumatic disorders look like but have not experienced trauma themselves react to stimulus words. The researchers trained a group of 31 persons who in self-report tests were able to get a diagnosis of PTSD. Thus, the participants of the study proper were: persons with PTSD after a road traffic accident (*N* = 35), persons simulating PTSD (*N* = 31), and a control group (*N* = 28). The average interference result also differed between persons simulating PTSD and those from the control group. No statistically significant differences were found between the group with PTSD and the group simulating PTSD in their reaction times to words pertaining to a road traffic accident. The work of Cisler et al. (2011) was published summing up research conducted with the use of the emotional Stroop test on groups of patients with PTSD. The meta-analysis concerned 26 studies conducted from 1990 to 2007. In total, 538 persons with PTSD, 274 persons after a trauma but without diagnosed PTSD, and 254 persons from control groups participated in the studies. The general conclusion from the meta-analysis is that the interference effect occurs in response to words related to the type of trauma experienced by persons with PTSD and in persons after a trauma but without PTSD compared with the control group. The differentiator is the fact of having experienced a trauma and not the presence of post-traumatic disorders.

The emotional Stroop test enables the identification of an important type of interference whose control depends on the properties of the emotion. The core area of the application of the test are clinical studies to assess – based on the interference degree indicators for words of various content – the degree of emotional disorders resulting from the trauma experienced by a person or the source of the trauma. A word related to the source of the trauma attracts the attention to a greater extent and thus it takes longer to name the font color (Williams et al., 1996). Undoubtedly, results of studies conducted with the use of the Stroop test and dot-probe task (van Rooijen et al., 2017) confirm the existence of anxiety influence on the processing of stimuli associated with threatening stimuli at an early stage of information processing.

To recapitulate, there is agreement among researchers as to the use of the emotional Stroop test to diagnose the tendentiousness of the attention functioning with regard to threatening stimuli associated with trauma. There is no consensus as to whether what occurs is a sensitization to a determined type of stimuli and hence the engagement of attention at the initial stage of processing, or the tendentiousness concerns a deeper analysis of stimuli (Song et al., 2017). Is therefore the tendentiousness of processing under the influence of anxiety linked to the blocking of further processing of the information as far as to the semantic level? Or is it about tendentious remembering of the material which was the first to engage attention resources? In the study presented in this article, the authors used a modified Stroop task, which will partially enable provide answers to these questions. We adopt the assumption that the vigilance/avoidance hypothesis may be verified by introducing a second series after the administration of the Stroop test proper, namely a series of stimuli recalling. The modification of the procedure enables verification of the assumption on the depth of processing of anxiogenic stimuli. The aforementioned studies, which monitored explicit concentration on stimuli, showed that from a certain moment participants diverted their attention from the stimulus, which indirectly verified the hypothesis on the difficulty in un-engaging and avoiding deeper processing. If that is the case, a comparison of color naming times for threatening words with the accuracy of their recalling will be a verification of the hypothesis on the level of processing depth. The authors previously quoted the results of a study in which free recalling was requested. However, the researchers did not compare the reaction times to words in the emotional Stroop test with the accuracy of recalling. In our study, prepared on computer, it is possible to precisely compare the reaction times with the accuracy of recalling the words used in the test. Accordingly, the author’s subjected to verification of three main research hypotheses:

(1) In post-traumatic persons there is a significantly stronger interference effect in reaction to negative words.
(2) The longer the reaction time to words associated with trauma, the less accurate the recalling of negative words in the group of post-traumatic persons.
(3) The higher the anxiety as a state in the studied group, the stronger the interference effect in the group of victims; in the group of perpetrators it is the opposite: the lower the declared level of anxiety, the higher the interference effect and the less accurate remembering of stimulus words associated with trauma.

## Materials and Methods

This study was carried out in accordance with the recommendations of the SWPS University of Social Sciences and Humanities Ethics Committee with written informed consent from all participants. All procedures performed in studies involving human participants were in accordance with the ethical standards of the Institutional and/or National Research Committee and with the 1964 Helsinki declaration and its further amendments or comparable ethical standards. Number of consent issued by the Ethics Committee: 7/I/10-11 from 23 November 2010. The research encompassed 157 individuals (a group of victims *N* = 47 and perpetrators *N* = 43 of road traffic accidents *N* = 90; age *M* = 32.81; *SD* = 9.85; control group *N* = 67; age *M* = 34.20, *SD* = 11.16). There were 17 women and 26 men in the group of perpetrators; 25 women and 22 men in the group of victims. There were 34 women and 33 men in the control group. The participants, tested after road traffic accidents, were patients of traumatology and orthopedic wards who had been involved in a road traffic accident up to a month prior to the research. The participants who had been involved in accidents are drivers regularly using their vehicles. The studies were conducted in hospitals in Warsaw. The heads of traumatology and orthopedic wards where the studies took place had consented to the research.

### Methods

#### Sociodemographic Questionnaire

The purpose of the questionnaire was to collect key information about the participants. The questionnaire contained questions about age, gender, education, time for which the person had held a driving licence, nature and frequency of car use, category of driving licence held, status in the accident, injuries sustained, date, and nature of the accident.

#### STAI – State–Trait Anxiety Inventory

The Polish STAI is an adaptation of the American STAI test. It was developed by Spielberger et al. (1983). The authors of the Polish adaptation of the test are C. D. Spielberger, J. Strelau, M. Tysarczyk, K. Wrześniewski (Sosnowski et al., 2006). The STAI questionnaire contains 40 statements, which are divided into two independent parts. The first part consists of 20 statements and tests the level of anxiety as a current emotional state. This part is extremely sensitive and may be used to check changes in mood even at short intervals. Meanwhile, the second part, which also consists of 20 statements, concerns anxiety understood as a personality trait. The research participant selects one of four answers. In the first part: decidedly not, rather not, rather yes, decidedly yes. In the second part: almost never, sometimes, often, almost always. The answers are added together giving an individual’s the level of anxiety which is based on the points obtained. The score values for each part of the questionnaire may range from 20 to 80 points. High scores determine a higher level of anxiety. An analysis of reliability was made using Cronbach’s alpha method. The alpha of state-anxiety indicator is 0.94 and of trait-anxiety 0.89.

#### Computerized Task – Modified Emotional Version of the Stroop Test

The emotional Stroop test, prepared with the use of SuperLab 4.0, was used to study the functioning of selective attention. The participant’s task is to react as quickly as possible to the color in which the stimulus word appears on the computer screen. The study consists of the following stages: (a) instructions to the trial part; (b) trial test; (c) instructions to the proper part; (d) main test; (e) instructions to the series with word recalling; (f) series with word recalling; (g) thanks. After the trial series, the following instructions appear on the screen: *In a moment you will see a few words. They will be displayed one by one and presented in various colours. Read carefully each word on the screen and wait for the next screen, on which colour names will be given. Your task is to say in what colour the word was presented. The left button means the colour on the left side and the right button is the colour on the right. Try to remember as many words as possible because you will be asked to recall them later on during the study. Please respond as quickly as possible!!! To start press “SPACE”…*

Sixteen stimuli with appropriate degrees of negativity, neutrality, and frequency of appearance in language selected by competent jurors are displayed successively. After this series the participant moves on to the next task, which is the series with recalling. Each participant is informed, before starting to perform the task, that there will be such a part. This part begins with the instructions displayed on the screen: *In a moment you will see individual words and your task will be to say whether a given word appeared in the previous stage of the study or not. The left button of the mouse means YES. The right button of the mouse means NO. Please respond as quickly as possible!!! To start press “SPACE”..*. This part presents all stimuli which appeared in the series proper as well as 16 buffer stimuli, that is eight neutral and eight associated with trauma. At the end, thanks for participation in the study are displayed.

### Analysis of the Results Obtained

A statistical analysis to test the hypotheses put forward was made in IBM SPSS Statistics, version 24. Key descriptive statistics was analyses with the use of the software, which made it possible to study the distributions of successive measured variables. Parametric tests were performed on all variables because skewness values did not exceed the conventional absolute value equals 2. The hypotheses were tested with the use of a series of correlation analyses, multivariate analysis of variance, regression analyses using a stepwise method of entering predictors into the model, and mediation analyses with the use of the A. F. Hayes PROCESS macro (2013). The significance level was adopted at the classic threshold of α = 0.05.

### Selective Attention Disorders in Victims and Perpetrators of Road Accidents

The verification of the hypothesis was based on an analysis of differences in reaction times to words associated with a traumatic event. The longer the reaction time to the stimulus words, the stronger the interference effect. The analysis was first made for two groups: tested and control. Subsequently, a division of the participants into three groups was taken into account: control, victims, and perpetrators. A two-factor variance analysis in a mixed model was conducted in order to verify the hypothesis on the influence of belonging to a particular group in the size of the interference effect. However, prior to proceeding with the analysis, outliers and so-called deviant observations were eliminated since such results might significantly influence the average reaction times of the compared groups. In practice this means removing observations above or below 2.58 standard deviations from the mean. Table 1 shows the descriptive statistics and intergroup comparisons.

**TABLE 1:** Descriptive statistics and intragroup, intergroup comparisons.

	**Road traffic accidents survivors**		**Control group**		***F***	***p*-value**
	***M***	***SD***	***M***	***SD***		
Age	34.01	10.77	34.20	11.16	*0.011*	*0.915*
Reaction time on the negative and neutral stimuli	940 ms	242 ms	849 ms	177 ms	*9.216*	***<0.05***
Reaction time on negative stimuli	981 ms^1^	252 ms	862 ms	197 ms	*11.022*	***<0.001***
Correct answers on negative stimuli	93.5%^2^	5%	97.88%	3.75%	*9.368*	***<0.001***
Reaction time on neutral stimuli	932 ms^1^	241 ms	836 ms	180 ms	*4.695*	***<0.05***
Correct answers on neutral stimuli	96.88%^2^	5%	97.13%	5%	*0.135*	*<0.712*
Reaction time on negative stimuli in recall series	1,410 ms	379 ms	1,214 ms	412 ms	*9.147*	***<0.001***
Correct answers on negative stimuli in recall series	76.75%	16%	86.87%	13%	*16.371*	***<0.001***
Anxiety-state	45.33	10.63	31.57	6.27	*84.630*	***<0.001***
Anxiety-trait	46.88	6.92	44.60	6.61	*3.761*	*0.055*

	**Perpetrators**		**Victims**			
	***M***	***SD***	***M***	***SD***	***F***	***p***

Age	32.81	9.85	35.16	11.57	1.040	0.311
Reaction time on the negative and neutral stimuli	981 ms	258 ms	903 ms	247 ms	*6.579*	***<0.05***
Reaction time on negative stimuli	1,041 ms^3^	289 ms	925 ms	289 ms	*5.361*	***<0.05***
Correct answers on negative stimuli	92.38%^4^	6%	95.13%	6%	*1.061*	*0.306*
Reaction time on neutral stimuli	921 ms^3^	260 ms	880 ms	220 ms	*1.190*	*0:279*
Correct answers on neutral stimuli	97.5%^4^	5%	97.25%	6%	*0.227*	*0.635*
Reaction time on negative stimuli in recall series	1,322 ms	268 ms	1,491 ms	445 ms	*4.338*	***<0.05***
Correct answers on negative stimuli in recall series	77%	16%	76.62%	17%	*0.016*	*0.900*
Anxiety-state	44.67	11.23	45.96	10.13	*0.321*	*0.573*
Anxiety-trait	47.95	4.48	45.84	8.61	*1.747*	*0.190*

*The results of the statistical test ANOVA are marked with italic. Significant results are marked with bold. ^1^Intra-group comparison of reaction times for RTA survivors *F*(1,143) = 21.054; *p* < 0.01, η^2^ = 0.123. ^2^Intra-group comparison of percentage of correct answers for RTA survivors *F*(1,151) = 19.927; *p* < 0.01, η^2^ = 0.123. ^3^Intra-group comparison of reaction times for perpetrators *F*(1,80) = 18.757; *p* < 0.001, η^2^ = 0.19. ^4^Iintra-group comparison of percentage of correct answers for perpetrators *F*(1,85) = 13.372; *p* < 0.01, η^2^ = 0.136.*

A statistically significant main effect of the intragroup factor was obtained *F*(1,143) = 16.995; *p* < 0.01. Moreover, a significant result of the main effect of the intergroup variable was also obtained *F*(1,143) = 9.216; *p* < 0.01. Regardless of the stimulus type, individuals from the tested group took longer to react *M* = 940 ms than participants from the control group *M* = 849 ms.

The next stage was an analysis of simple main effects, since a significant effect of the interaction between the intergroup and intragroup factors was obtained *F*(1,143) = 4.280; *p* < 0.05. A statistically significant simple main effect for negative stimuli was obtained *F*(1,143) = 11.022; *p* < 0.001, respectively, the tested group had an average time *M* = 981 ms and the control group *M* = 862 ms. In the case of simple main effects for neutral stimuli there was also a statistically significant difference *F*(1,143) = 4.695; *p* < 0.05, but it is lower than in the case of words associated with the accident. Also, a significant simple main effect of the intragroup factor was found in the tested group. The reaction times to neutral words are significantly longer than reaction times to negative words *F*(1,143) = 21.054; *p* < 0.01. In the control group, a simple main effect of the intragroup factor is insignificant *F*(1,143) = 1.935; *p* = 0.166. These results emphasize the specific nature of reacting in the tested group, since irrespective of the value of the average reaction times the persons who had been involved in accidents react longer to words associated with the accident than to neutral stimuli.

Additionally, using only a division into tested and control groups, the accuracy of the reaction to the stimulus words was checked. A two-factor variance analysis in a mixed model was conducted. A statistically significant main effect of the intragroup factor was shown *F*(1,151) = 4.661; *p* < 0.05. Regardless of their belonging to a particular group, participants more often reacted correctly to neutral words *M* = 97.25% than to negative ones *M* = 95.87%. The main effect of the intergroup factor is insignificant *F*(1,15) = 2.683; *p* = 0.104. Moreover, a significant effect of the interaction between the intragroup and intergroup factors was shown *F*(1,151) = 13.726; *p* < 0.01. The simple main effect for the correctness of reaction to negative stimuli is statistically significant. The differences between the tested and control groups in the number of correctly named font colors of the negative words in the proper series are significant *F*(1,151) = 9.368; *p* < 0.001. The simple main effect for the correctness of reaction to neutral stimuli is insignificant *F*(1,151) = 0.135; *p* = 0.712. It is also worth noting here that individuals from the tested group needed significantly more time to respond to the question about the color in which the word was presented and in spite of that made more errors.

The next step was an analysis of simple main effects of the correctness of the reaction separately for the tested and control groups. It was shown that in the tested group the correctness of the reaction to neutral words was significantly higher than the correctness of the reaction to negative words *F*(1,151) = 19.927; *p* < 0.01. In the control group the difference is insignificant *F*(1,151) = 1.051; *p* = 0.307. Once again it turns out that in the tested group we obtain a significant difference in the correctness of the reaction to the color of a negative word compared with the neutral stimulus. The result obtained confirms the hypothesis concerning the difficulty in un-engaging attention from threatening words, and thereby it permits the statement that in the tested group there are deficits in executive control. Participants from the group of those who had been involved in accidents find it hard to switch from automated to conscious functioning and reflectively process stimuli and perform complex cognitive operations. The lack of cognitive resources results from being absorbed in the meaning of the threatening stimulus.

The above explanations are corroborated by the *r*-Pearson correlation tests. A significant correlation was shown between the number of correctly named colors of words associated with the accident and the average reaction time for the tested group *r* = −0.340; *p* < 0.00. The longer the decision-making process lasted, the more errors were made. In the control group no significant correlation was shown *r* = −0.006; *p* = 0.964. Moreover, a significant correlation was found between the number of correctly named colors of words unrelated to the accident and the average reaction time for the tested group *r* = −0.269; *p* < 0.001. As in the case of negative words, the longer decision-making time in the tested group was correlated to a smaller number of correct answers. In the control group, no significant correlation was shown *r* = −0.106; *p* = 0.399. The authors then proceeded to check whether the results obtained for the tested group considered as a whole also occur when we then into groups of perpetrators and victims. A comparative analysis between the groups was undertaken once again in order to verify the hypothesis on the size of the interference effect.

**FIGURE 1:**
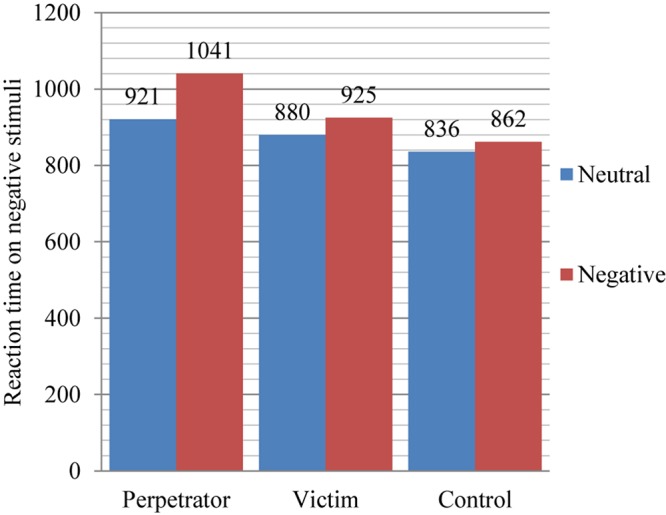
Differences in the interference effect between the groups of perpetrators, victims, and the control group to neutral and negative stimuli in the series proper (for details see Table 1).

The same variance analysis was performed as for the division only into the tested and control groups. A statistically significant main effect of the intragroup factor was shown *F*(1,142) = 23.255; *p* < 0.001. Regardless of their belonging to a particular group, participants had longer reaction times to negative stimuli *M* = 943 ms than to neutral ones *M* = 879 ms. The main effect of the intergroup factor is also statistically significant *F*(2,142) = 6.579; *p* < 0.01. Irrespective of the stimulus type, individuals from the group of perpetrators have significantly longer reaction times *M* = 981 ms than the control group *M* = 849 ms. The participants from the group of victims *M* = 903 ms and from the control group do not differ in their general average reaction times. Besides the above two main effects, a significant effect of the interaction between the intragroup and intergroup factors was obtained *F*(2,142) = 4.531; *p* < 0.05 and for this reason simple main effects were analyzed. A statistically significant result was obtained in the case of differences in the reaction times to the words associated with the accident *F*(2,142) = 8.693; *p* < 0.001, respectively, the group of perpetrators had an average time *M* = 1041 ms, the group of victims *M* = 925 ms, and the control group *M* = 862 ms. Additional multiple comparisons with the Sidak correction showed differences between the group of perpetrators and the control group as well as between the group of perpetrators and that of victims. The results obtained are presented in Figure 1.

### Reaction Time and Recognition Correctness

**FIGURE 2:**
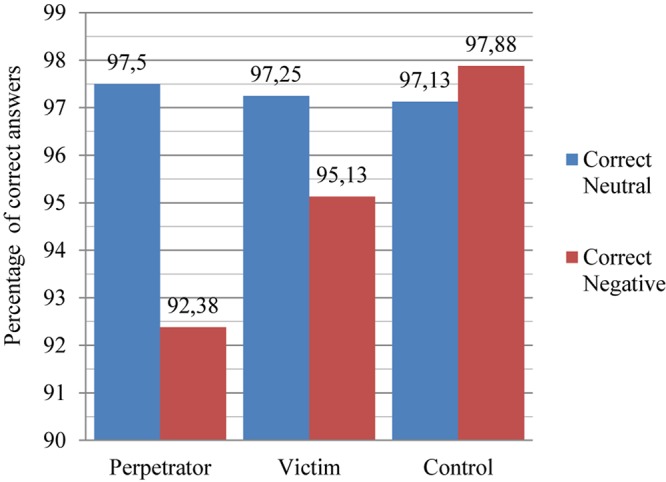
Differences between the groups in terms of correctness of the responses (naming word color) to negative and neutral stimuli (for details see Table 1).

A check of the relationship between the length of reaction times and the number of correctly named colors of words associated with the accident showed a significant negative correlation *r* = −0.355; *p* < 0.001. The existence of difficulties in switching attention from negative stimuli processing and difficulties in inhibiting negative stimuli processing may be stated in the group of perpetrators. No such correlation was found between the number of correctly named colors of words unrelated to the accident and the average reaction time for the group of perpetrators *r* = 0.268; *p* = 0.094. The lack of such a correlation in the case of neutral stimuli all the more justifies the thesis about the existence of attention deficits in the event of processing negative stimuli. A similar correlation was found in the group of victims: *r* = −0.333; *p* < 0.05. The longer the decision-making time, the more errors are made. Furthermore, in this group a significant correlation was shown between the number of correctly named colors of words unrelated to the accident and the average reaction time for the group of victims *r* = −0.591; *p* < 0.001. In this group there is excessive vigilance, which translates into treating neutral words as potentially threatening. It can be concluded that the diffuse negative affect had been generalized onto the processing of neutral stimuli. It is worth asking at this point to what extent the observed regularities are temporary and do not become consolidated, and what is conducive to such a consolidation. The results obtained are presented in Figure 2.

### Anxiety as a Predictor of Selective Attention Disorders

Linear regression analysis was made in order to check the influence of anxiety as a state on reaction time (size of the interference effect) to the words associated with the accident. The analyses were made separately for the group of perpetrators and the group of victims. It transpired that in the group of perpetrators the model is well suited to the data *F*(1,38) = 4.434; *p* < 0.05 and explains 10.4% of the variance of the results for the dependent variable. Anxiety as a state is a strong negative predictor (beta = −0.323). The lower the declarative level of anxiety, the longer the reaction time to words associated with the accident in the group of perpetrators. This result may mean that on the declarative level the perpetrators of accidents experience a lower level of anxiety or they do not interpret the symptoms of the physiological state as anxiety. However, on the implicit level they experience strong anxiety, which was reflected in the longer reaction times. It is worth noting here that such a discrepancy may result in many negative consequences in the future such as the willingness to confirm one’s good driving skills by making a lot of risky decisions while driving just to prove oneself. A similar analysis was performed for the group of victims. It appeared that in the group of victims the model is well suited to the data *F*(1,43) = 6.419; *p* < 0.05 and explains 13% of the variance of the results for the dependent variable. Anxiety as a state is a strong positive predictor (beta = 0.360). The higher the level of anxiety, the longer the reaction time to words associated with the accident in the group of victims. This result shows the consistency in the functioning of victims of accidents. Being a victim, one may give oneself the right to experience anxiety related to a difficult situation in which one was put, since there is no fault of the victim in this and there is no need to use mechanisms protective of the ego. The analysis shows that both awareness of experienced anxiety and the unconscious experience of anxiety significantly extend reaction time, thereby increasing interference.

**FIGURE 3:**
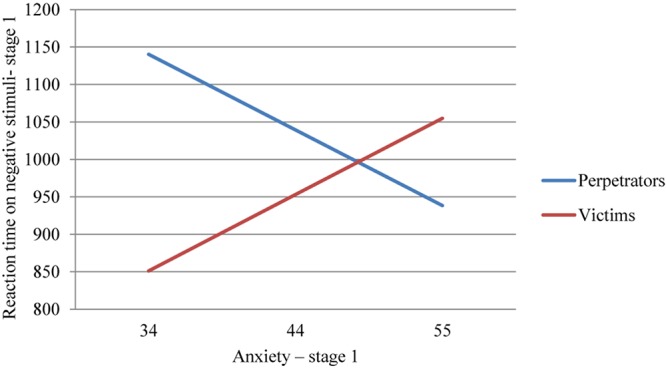
The moderation effect of the role in accident on the relation between anxiety and reaction time on negative stimuli.

A macro developed by Hayes (2013), “PROCESS,” was used to assess whether being a perpetrator or victim is a moderator of the relationship between the level of anxiety and reaction time. A statistically significant model was obtained [*F*(3,73) = 6.08; *p* < 0.001], which explains 20% of the reaction time variance. It was observed that a longer reaction time depends on a lower level of anxiety; moreover, the reaction times were longer in the group of perpetrators (*p* < 0.001). Also, moderation by the group was also confirmed in the case of correlation between anxiety and reaction time (*p* < 0.001). A detailed analysis of conditional effects showed that an interaction was observed both in the group of perpetrators (*p* < 0.001) and in the group of victims (*p* < 0.01). In the group of perpetrators, the reaction time increases as the felt level of anxiety diminishes (Coeff = −9.49). In the case of victims, the opposite relation was obtained (Coeff = 9.58). The results obtained are presented in Figure 3.

### Level of Anxiety and the Depth of Coding of Threatening Cognitive Content

**FIGURE 4:**
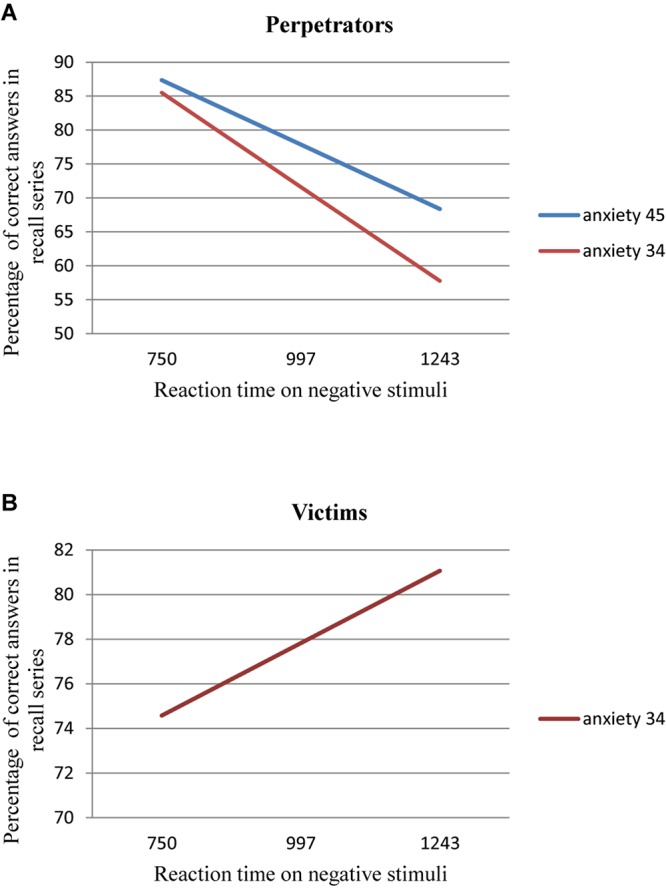
**(A,B)** The moderation effect of the anxiety on the relation between the percentage of the correct answers in recall series and reaction time on negative stimuli.

A measure of the depth of coding in the study is the number of correctly recalled words from the phase proper of the experiment (in the Stroop task). Based on attention functioning analyses measured by explicit measures, the authors assumed that persons who had been involved in road traffic accidents would remember fewer words than persons who had not been involved in road traffic accidents and would need more time to say whether a given word appeared earlier in the test or not. For this purpose, we had to examine whether any differences between groups existed in the number of correctly recalled words. The accuracy of recalling negative words from the test proper was checked. A variance analysis in the intergroup model showed a statistically significant difference between the tested and control groups in the number of correctly recalled negative words, *F*(1,152) = 16.371; *p* < 0.001. Persons who had been involved in road traffic accidents correctly qualified on average *M* = 76.75% words and the participants from the control group *M* = 86.87% words. Persons from the tested group needed significantly more time to answer the question whether a given word appeared previously in the test or not *F*(1,148) = 9.147; *p* < 0.001, respectively, the tested group *M* = 1,410 ms while the control group *M* = 1,214 ms. In order to perform an in-depth analysis, the macro developed by A. F. Hayes, “PROCESS” was used to assess whether the reaction time to the negative words in the series proper influenced the number of correctly recalled words in the following series. Additionally, the model took into account the level of anxiety as a state (mediator) and being a perpetrator or victim (moderator). A significant model was obtained *F*(7,73) = 3.99; *p* < 0.05, which explains 16% of the variance of the explained variable. It appeared that significant predictors in the model are the interaction of the reaction time to the negative words in the series proper × anxiety as a state (*p* = 0.0207) and moderated mediation of being a perpetrator or victim, reaction time, and anxiety as a state (*p* = 0.0265). For perpetrators with low (*p* = 0.019) and medium (*p* = 0.018) levels of anxiety, the longer the time, the fewer correct answers. For victims with low (*p* = 0.043) level of anxiety there is an opposite relation, the longer the time, the more correct answers. The analyses are presented in Figures 4A,B.

## Discussion

Research by Calvo and Avero (2002) with the use of the method of continuous measuring of eye movement showed that attention processing in highly anxious persons proceeds as follows: careful search of the attention field at the beginning of exposure, spotting anxiety-causing elements, and subsequently a perceptual rejection of the stimulation to prevent cognitive overload and “affective flooding.” The stimulation coded at the initial stage is then coded at a deeper level, thereby engaging attention resources and causing attention to be distracted from subsequent anxiety-causing elements. Undoubtedly, when a stimulus is directly associated with a personally difficult experience, the rejection phenomenon may occur faster and signal an activation of a personal network of associations or, as Foa puts it, network of fear (Foa and Kozak, 1986, 1993; Foa and Cahill, 2001). Macintosh and Hoppitt (2009) attribute the tendentiousness of the attention to the operation of an individual’s motivational processes related to the person’s plans and goals. According to researchers, convictions about oneself in conjunction with convictions concerning actions and their planning are responsible for the tendentiousness of the attention. As a result of these assumptions, the interference in the emotional Stroop test is subject to the action of a volitionally activated program whose purpose is to seek any negative stimuli that might appear (Macintosh and Hoppitt, 2009). When the individual encounters such stimuli, the person may get “anchored” on them, especially when monitoring was previously preceded by the feeling of anxiety.

According to our knowledge, this is the first studies focusing on the new version of the emotional Stroop test that enables a study of the process of the depth of coding of the stimuli associated with trauma. Longer reaction times to threatening words in the emotional Stroop test indicate a strong concentration on the meaning of the word, and full engagement of attention resources. The results obtained in this study prove that in the groups of both victims and perpetrators of road traffic accidents within a period of up to 1 month after the event the speed of processing the information associated with the past trauma is significantly reduced. The time that has elapsed since the event strongly influences cognitive functioning and indeed it can be said that the suddenness of the event and lack of control over what is happening to us impact the depth of memory trace consolidation. Our results confirm the hypothesis on the vigilant/avoidant style of information processing. As the authors previously mentioned, consolidation of a trauma in memory may be reflected in the form of increased availability of a cognitive representation, converting it into a heuristic used for interpretation. The most salient hypothesis in the presented research is one pointing to attention disorders that consist in problems with diverting the attention from the processing of a threatening stimulus, which was reflected, among others, in the poorer recalling of negative words and in a greater number of errors made in recognizing the colors of negative words. If we consider the fact that the tested individuals were a few days after the accident, it is justified to explain the results obtained with reference to an excessive vigilance characteristic of post-traumatic persons. While at this moment after the traumatic event an excessive vigilance may be treated as a correct reaction, with reference to the previously quoted fear network model of Foa and collaborators, it can be assumed that the maintenance of such reactions for a longer time after the event may be a symptom of, among others, the development of a pathological fear network.

Another interesting result are the differences between the tested group (victims and perpetrators) and the control group in terms of average reaction times to both negative and neutral stimuli. In the tested group there is additionally a negative correlation between the reaction time to words associated with the accident and the number of correctly recalled words. The longer the perpetrators and victims of accidents looked at the words, the fewer words they remembered. The results of the study also show that the status the person has in the accident is relevant. In the case of victims, based on their declarative levels of anxiety, longer reaction times may be predicted. In the case of perpetrators, lower scores on their declared levels of anxiety are accompanied by longer reaction times to negative stimuli. This might be the result of using mechanisms protective of the ego in order to diminish the importance of the event in which the person was the perpetrator. Besides, as followed from earlier analyses, perpetrators experienced anger more intensely (Ścigała, 2013) and on the conscious level this emotion may “cover up” anxiety. Underestimation of anxiety in perpetrators may result in selective attention disorders in natural situations. Denial of anxiety at a conscious level and, as follows from the research, experiencing it at an unconscious level result in evident disorders of cognitive control. The authors previously quoted the results of research on brain activity in the emotional Stroop test, which indicated that the stronger the interference effect, the greater the dysfunction of the prefrontal cortex (Borkowska, 2006; Coates, 2008; Liu et al., 2015; Song et al., 2017). Quick and accurate decisions are sometimes required from drivers in dangerous situations on the road. The results obtained show that underestimating one’s own anxiety results in a worse functioning of cognitive processes, overestimating one’s own abilities and, if a measure of the riskiness of a situation is the individual’s ability to draw conclusions about one’s own arousal, perpetrators evidently assess their arousal inaccurately.

To recapitulate, it may be said that the longer the tendentiousness in negative stimuli processing is maintained, the greater probability of the disorder getting consolidated. Such a state may constitute serious constraint to efficient movement on the roads and functioning in general. The longer the anxiety is maintained at either an explicit or implicit level, the more the fear network spreads, connecting increasingly more stimuli, reactions, and meanings. The interference effect in the Stroop test enables a rapid and accurate identification of the size of cognitive deficits in victims and perpetrators of accidents. What is more, studying deficits in attention processes and more advanced executive control in the group of persons who were involved in road traffic accidents may constitute an important diagnostic instrument of the individual’s cognitive ability to be an active participant of road traffic.

## Conclusion

To summarize, our results point toward the importance of the role in road traffic accident as a variable moderating the presence of emotional Stroop effect in patients. The research carried out is based on the conclusion that self-report methods to study the effects of trauma-related experiences are accurate in relation to only for the victims of road accidents. However, there is a definitely different pattern in the case of the perpetrators of accidents. Examination of the level of anxiety using a questionnaire does not reflect the essence of its intensity. The results show that the use of not self-reports research methods measure negative emotions, as anxiety in this group is much more accurate. The perpetrators who do not admit to the intensity of anxiety can actually be misleading themselves and the professionals from the psychological health. Concerning the exploratory nature of the study, reported results certainly require independent replication and also allow to determine to what extent our findings generalize to other clinical populations, for example, victims and perpetrators with PTSD and different versions of emotional Stroop test.

### Limitations

Our study certainly requires further validation. If such efforts were successful, the conclusions would bear important implications for the understanding of cognitive biases in coding threatening stimuli. Longitudinal research will allow indicating which measure of anxiety disorder may be a better predictor of PTSD in victims and perpetrators of accidents.

## Ethics Statement

All procedures performed in studies involving human participants were in accordance with the ethical standards of the Institutional and/or National Research Committee and with the 1964 Helsinki declaration and its later amendments or comparable ethical standards. The study was reviewed and approved by the SWPS University of Social Sciences and Humanities Ethics Committee. Number of consent issued by the Ethics Committee: 7/I/10-11 from 23 November 2010.

## Author Contributions

DŚ and EZ-Ś made substantial contributions to the conception or design of the work or the acquisition, interpreted the data for the work, drafted the work or revised it critically for important intellectual content, approved the final version to be published, and agreed to be accountable for all aspects of the work in ensuring that questions related to the accuracy or integrity of any part of the work are appropriately investigated and resolved. DŚ prepared the experimental procedure programming, experiment execution, and analyzed the data.

## Conflict of Interest Statement

The authors declare that the research was conducted in the absence of any commercial or financial relationships that could be construed as a potential conflict of interest.
